# Reprogramming Effects of Postbiotic Butyrate and Propionate on Maternal High-Fructose Diet-Induced Offspring Hypertension

**DOI:** 10.3390/nu15071682

**Published:** 2023-03-30

**Authors:** You-Lin Tain, Chih-Yao Hou, Guo-Ping Chang-Chien, Sufan Lin, Hong-Tai Tzeng, Wei-Chia Lee, Kay L. H. Wu, Hong-Ren Yu, Julie Y. H. Chan, Chien-Ning Hsu

**Affiliations:** 1 Department of Pediatrics, Kaohsiung Chang Gung Memorial Hospital, Kaohsiung 833, Taiwan; tainyl@cgmh.org.tw; 2 Institute for Translational Research in Biomedicine, Kaohsiung Chang Gung Memorial Hospital, Kaohsiung 833, Taiwan; 3 College of Medicine, Chang Gung University, Taoyuan 330, Taiwan; 4 Department of Seafood Science, National Kaohsiung University of Science and Technology, Kaohsiung 811, Taiwan; chihyaohou@webmail.nkmu.edu.tw; 5 Institute of Environmental Toxin and Emerging-Contaminant, Cheng Shiu University, Kaohsiung 833, Taiwan; guoping@csu.edu.tw (G.-P.C.-C.); linsufan2003@csu.edu.tw (S.L.); 6 Super Micro Mass Research and Technology Center, Cheng Shiu University, Kaohsiung 833, Taiwan; 7 Center for Environmental Toxin and Emerging-Contaminant Research, Cheng Shiu University, Kaohsiung 833, Taiwan; 8 Department of Urology, Kaohsiung Chang Gung Memorial Hospital, College of Medicine, Chang Gung University, Kaohsiung 833, Taiwan; 9 Department of Pharmacy, Kaohsiung Chang Gung Memorial Hospital, Kaohsiung 833, Taiwan; 10 School of Pharmacy, Kaohsiung Medical University, Kaohsiung 807, Taiwan

**Keywords:** butyrate, developmental origins of health and disease (DOHaD), fructose, gut microbiota, hypertension, propionate, short-chain fatty acid

## Abstract

Maternal nutrition has a key role in the developmental programming of adult disease. Excessive maternal fructose intake contributes to offspring hypertension. Newly discovered evidence supports the idea that early-life gut microbiota are connected to hypertension later in life. Short-chain fatty acids (SCFAs), butyrate, and propionate are microbiota-derived metabolites, also known as postbiotics. The present study aimed to determine whether maternal butyrate or propionate supplementation can protect offspring from hypertension using a maternal high-fructose (HF) diet rat model. Female Sprague Dawley rats were allocated during pregnancy and lactation to (1) regular chow (ND); (2) 60% high-fructose diet (HF); (3) HF diet plus butyrate (HFB, 400 mg/kg/day); and (4) HF diet plus propionate (HFP, 200 mmol/L). Male offspring were sacrificed at 12 weeks of age. The maternal HF diet impaired the offspring’s BP, which was prevented by perinatal butyrate or propionate supplementation. Both butyrate and propionate treatments similarly increased plasma concentrations of propionic acid, isobutyric acid, and valeric acid in adult offspring. Butyrate supplementation had a more profound impact on trimethylamine N-oxide metabolism and nitric oxide parameters. Whilst propionate treatment mainly influenced gut microbiota composition, it directly altered the abundance of genera *Anaerovorax, Lactobacillus*, *Macellibacteroides*, and *Rothia*. Our results shed new light on targeting gut microbiota through the use of postbiotics to prevent maternal HF intake-primed hypertension, a finding worthy of clinical translation.

## 1. Introduction

Maternal nutrition has substantial implications for fetal growth and development. Nutritional inadequacy or excess during pregnancy may alter the structure and function of the offspring’s organs permanently, resulting in various adult diseases through so-called developmental programming, or Developmental Origins of Health and Disease (DOHaD) [1,2]. In the past few decades, global consumption of fructose has increased and been associated with an increased risk of hypertension [3]. As the kidney is particularly sensitive to the effects of fructose, prior research indicated that a maternal high-fructose diet can impair kidney development, leading to programmed hypertension in adult offspring [4,5]. 

In contrast, the DOHaD theory affords an innovative strategy to avert hypertension through reprogramming—an approach that shifts therapeutic intervention from adulthood to the earliest stage of life, prior to hypertension occurring [6]. Nutritional interventions during pregnancy have lately started to attract attention as a reprogramming strategy to avert the developmental programming of hypertension [2]. 

Maternal nutrition influences the composition of fetal gut microbiota and thereby the health of offspring [7]. Previous research indicated that a high-fructose diet during pregnancy not only altered gut microbiota compositions and microbiota-derived metabolites in mother rats but also in their adult offspring [8]. Additionally, some gut microbiota-targeted therapies have been applied to avert maternal fructose diet-primed developmental programming [8]. 

Short-chain fatty acids (SCFAs) such as propionate, butyrate, and acetate are the main microbiota-derived metabolites [9]. Dietary fiber is fermented by the gut microbiota to produce SCFAs. Through activation of their receptors, SCFAs can directly regulate blood pressure (BP) [9]. Postbiotics are non-viable metabolic byproducts or bacterial products formed by probiotic microorganisms [10]. Acting as postbiotics, SCFAs have shown benefits for maternal health and fetal development [11]. Additionally, perinatal butyrate and propionate supplementation revealed their protective actions against offspring hypertension in a maternal tryptophan-free diet model and a maternal chronic kidney disease model, respectively [12,13]. Our prior work observed that maternal HFD-induced hypertension is also relevant to another microbial metabolite, trimethylamine (TMA) [14]. TMA can be converted into trimethylamine N-oxide (TMAO), which has been closely linked with the development of hypertension [15]. Although TMA inhibition has been shown to be beneficial for programmed hypertension [15], whether maternal SCFA supplementation can prevent maternal HF diet-induced offspring hypertension via regulation of the TMA-TMAO pathway is yet to be determined. Moreover, the aberrant renin-angiotensin system (RAS) and deficiency in nitric oxide (NO) also anticipate hypertension programming in various animal models, such as the maternal high-fructose diet model [16].

Hereafter, the objective of the present study is to assess whether perinatal supplementation with butyrate or propionate can prevent maternal high-fructose diet-primed offspring hypertension through alterations in the microbial compositions, microbiota-derived metabolites, and other mechanisms involved in the regulation of BP.

## 2. Materials and Methods

### 2.1. Design of Animal Protocol

The Kaohsiung Chang Gung Memorial Hospital Institutional Animal Ethics Committee granted ethical approval for this study (permit number: 2022011001). Virgin Sprague–Dawley (SD) rats were raised and bred in an animal facility accredited by AAALAC International. One female and one male were housed together for mating. A successful pregnancy was confirmed by the occurrence of copulatory plugs.

Pregnant SD rats received regular chow (ND, *n* = 3) or a 60% fructose diet (*n* = 9) during the pregnancy and lactation periods [4]. Some fructose-fed rats received sodium butyrate (400 mg/kg/day) or supplementation of drinking water with sodium propionate (200 mmol/L) during the periods of pregnancy and lactation. The doses of butyrate and propionate used here were adopted according to our previous rat studies [12,13]. 

At birth, litters were culled into eight pups. Only male progeny were used in the following experiments, as males have higher probabilities of developing hypertension than females [17]. Male progeny were allocated into four groups (*n* = 8 from three independent litters/group): ND (normal chow diet), HF (60% fructose diet), HFB (60% fructose diet plus butyrate supplementation), and HFP (60% fructose diet plus propionate supplementation). Pups were weaned by three weeks and on to normal chow.

Noninvasive BP measures in conscious retrained rats were done using the CODA tail-cuff system from Kent Scientific Corp. (Torrington, CT, USA). Each rat was placed in restraint holders and allowed to adapt to the procedure one week before the actual recording session. Rat offspring were sacrificed after 12 weeks. Ahead of the sacrifice, we collected fecal samples and kept them at −80 °C in a freezer until extraction. Both kidneys were excised and weighed. Blood was collected from rats and placed into heparin tubes. Plasma samples were aliquoted and stored at −80 °C in a freezer. Creatinine concentrations in plasma were measured using an HP series 1100 high-performance liquid chromatography (HPLC) system (Agilent Technologies, Wilmington, DE, USA).

### 2.2. GC–MS

Analysis of plasma SCFA concentrations was conducted using a gas chromatograph-mass spectrometer (GC-MS, Agilent Technologies) as we described previously [12,13]. Acetic acid, propionic acid, butyric acid, isobutyric acid, valeric acid, and isovaleric acid were determined. The injection volume was 1 µL with a split ratio of 5:1. The 2-ethylbutyric acid was utilized as an internal standard.

### 2.3. LC-MS

An analysis of the plasma concentrations of TMAO, TMA, and DMA (the metabolite of TMAO and TMA) was carried out using an Agilent 6410 Series Triple Quadrupole LC/MS [14]. Briefly, DMA, TMA, and TMAO were monitored from the multiple reaction monitoring in positive-ion mode using characteristic precursor-product ion transitions: m/z 46.1→30, m/z 60.1→44.1, and m/z 76.1→58.1, respectively. The mobile phase consisted of methanol (20:80, *v*/*v*) with phase A (15 mmol/L ammonium formate) and phase B (acetonitrile), using a flow rate of 0.3–1 mL/min.

### 2.4. 16S rRNA Sequencing and Metagenomics Study of Gut Microbiota

We extracted microbial DNA from the fecal samples. Metagenomic analysis using 16S ribosomal RNA genes was carried out (Biotools Co., New Taipei City, Taiwan) [13]. Amplification of the bacterial full-length 16S rRNA gene for PacBio sequencing (Menlo Park, CA, USA) was generated using barcode primers adapted for SMRTbell library preparation. Using the QIIME2 software package [18], all the downstream analyses on these sequences were performed. From the amplicon sequence variants (ASVs) sequences, a phylogenetic tree was constructed via FastTree (QIIME2). For the α-diversity analysis, the Shannon index and Faith’s phylogenetic diversity (PD) index were used. For the β-diversity analysis, we used the analysis of similarities (ANOSIM) and the principal coordinate analysis (PCoA) with unweighted UniFrac distance. Moreover, linear discriminant analysis effect size (LEfSe) was applied to identify the most differentially abundant taxa between groups. 

### 2.5. Quantitative PCR

Total RNA extraction from renal cortical tissue and real-time quantitative PCR (qPCR) were performed as we described previously [13]. SCFA-sensing G protein-coupled receptors (GPR), including GPR41, GPR43, GPR109A, and olfactory factor 78 (Oflr78), were analyzed. The components of the RAS included renin, angiotensin-converting enzyme-1 (ACE1), angiotensin II type 1 receptor (AT1R), ACE2, (pro)renin receptor (PRR), and MAS receptor (MAS) and they were analyzed. Each sample was run in duplicate. The 18S ribosomal RNA (R18S) was utilized as the reference gene to normalize qPCR data. Table 1 offers the primer sequences used for qPCR. To determine relative gene expression, the comparative threshold cycle (Ct) method was used. The fold change was computed by comparing the relative gene expression between the target and the control using the formula 2^−ΔΔCt^. 

### 2.6. NO-Related Parameters

A validated HPLC method was used for measurements of NO parameters, including substrate L-arginine and its methylated derivatives, asymmetric and symmetric dimethylarginine (ADMA and SDMA). We used HPLC with fluorescence detection of o-phthaldialdehyde (OPA)/3mercaptopropionic acid (3MPA) derivatives to measure L-arginine and its methylated derivatives [16]. Homoarginine was utilized as an internal standard. We calculated the L-arginine-to-ADMA ratio to denote NO bioavailability [19]. 

### 2.7. Statistical Analysis 

All data are presented as the mean ± standard error of means (SEM). A one-way analysis of variance (ANOVA) with Tukey post hoc was utilized to determine differences between groups. Statistical analysis was performed using SPSS version 17.0 (SPSS Inc., Chicago, IL, USA). A *p*-value of <0.05 indicated statistical significance.

## 3. Results

### 3.1. Anthropometrics and BP of Male Offspring at Week 12

**Figure 1:**
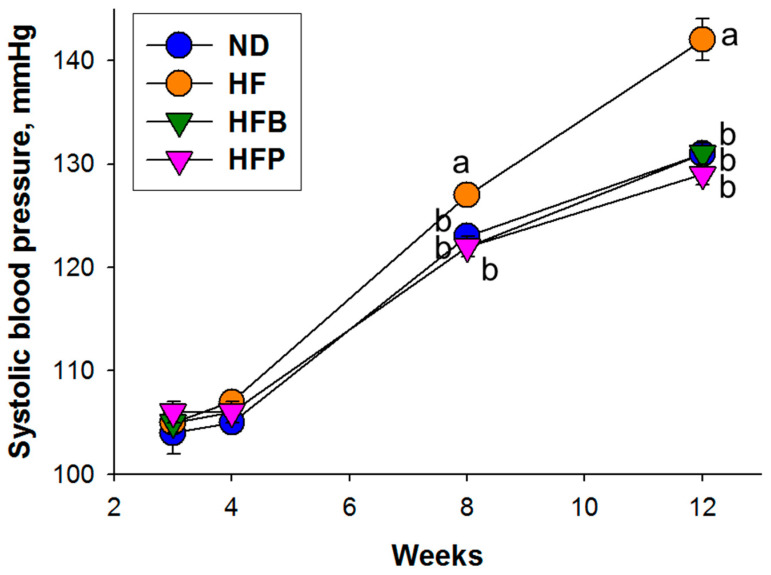
Systolic BP of male offspring at 3 to 12 weeks of age. *n* = 8/group; the letters represent the differences between the groups. Statistical analysis by a one-way ANOVA, *p* < 0.05.

No pup mortality was observed. At 12 weeks of age, male HF and HFB offspring had greater body weight (BW) gain in comparison to the ND and HFP offspring (Table 2). Similar patterns were seen with kidney weight (KW), while the HFP offspring displayed the lowest KW-to-BW ratio between the four study groups. Figure 1 reveals that a maternal high-fructose diet caused a rise in systolic BP from eight to 12 weeks. These increases in systolic BP induced by maternal HF intake were prevented by perinatal butyrate or propionate treatment. Similar patterns were observed with diastolic BP and mean arterial pressure at 12 weeks (Table 2). No differences in plasma creatinine concentration among the four groups of offspring were seen. 

In total, these data show that the maternal HF diet caused hypertension and BW gain in male offspring. Maternal HF diet-primed offspring hypertension was improved by butyrate or propionate treatment. 

### 3.2. Plasma SCFA Levels and Renal SCFA Receptors

Table 3 illustrates the impact of perinatal butyrate and propionate supplementation on plasma SCFA levels in offspring at age 12 weeks. Maternal HF diets remarkably reduced plasma levels of propionic acid, isobutyric acid, and valeric acid in the HF offspring compared with those in the ND offspring. Compared with HF offspring, HFB offspring had higher blood concentrations of propionic acid, isobutyric acid, butyric acid, and valeric acid. Similar patterns were seen with HFP offspring, except there was no difference in butyric acid levels between the HF and HFP groups. No differences in plasma concentrations of acetic acid and isovaleric acid among the four groups of offspring were seen. 

**Figure 2:**
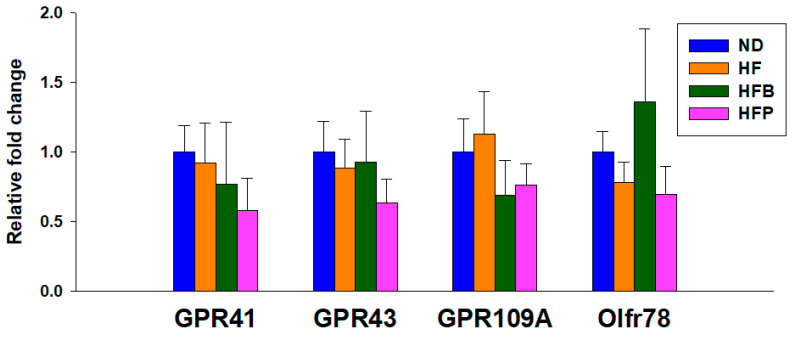
Renal mRNA expression of G protein-coupled receptor 41 (GPR41), GPR43, GPR109A, and olfactory receptor 78 (Oflr78) of male offspring at 12 weeks of age. *n* = 8/group.

Figure 2 shows that no differences in GPR41, GPR43, GPR109A, and Olfr78 expression in offspring kidneys among the four groups were seen.

### 3.3. TMAO, TMA, and DMA

**Figure 3:**
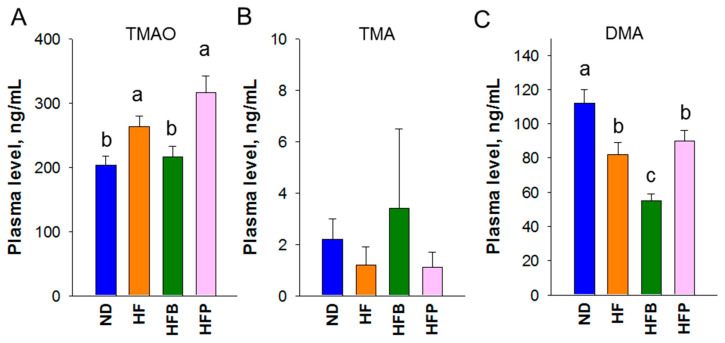
Plasma concentrations of (**A**) trimethylamine-N-oxide (TMAO), (**B**) trimethylamine (TMA), and (**C**) dimethylamine (DMA) of male offspring at 12 weeks of age. The letters represent the differences between the groups. *n* = 8/group. Statistical analysis by one-way ANOVA, *p* < 0.05.

HF offspring had a higher level of TMAO in the plasma than that in the ND group (Figure 3). This was accompanied by a lower plasma DMA level in HF offspring compared with ND. Maternal butyrate supplementation showed an improvement in TMAO but caused a further decrease in DMA in the HFB offspring. There was no difference in plasma TMA concentrations between groups (Figure 3B). 

### 3.4. Gut Microbiota Composition

**Figure 4:**
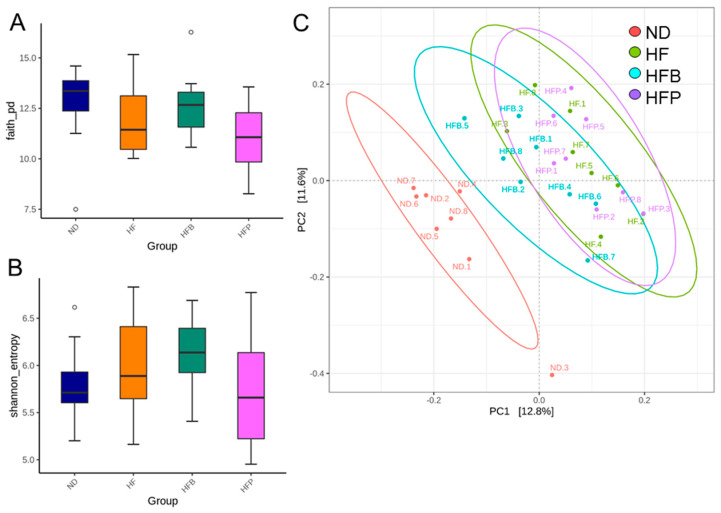
α-diversity measures: (**A**) Faith’s phylogenetic diversity (PD) index and (**B**) Shannon index. (**C**) β-diversity analysis using principal coordinate analysis (PCoA). Each dot denotes the microbiota for a given sample, and the dot color reveals the grouping for that sample. *n* = 8/group.

Microbial α- and β-diversity of rat offspring at 12 weeks of age are shown in Figure 4. A comparison of α-diversity measures revealed neither the Faith PD index (Figure 4A) nor the Shannon index showed a difference (Figure 4B). As shown in Figure 4C, β-diversity, based on an unweighted UniFraq distance matrix, was visualized by a PCoA plot, and four different clusters were defined. The ANOSIM analysis showed significant differences between almost all of the groups (*p* < 0.05), except the HF and HFB groups (*p* = 0.083). 

**Figure 5:**
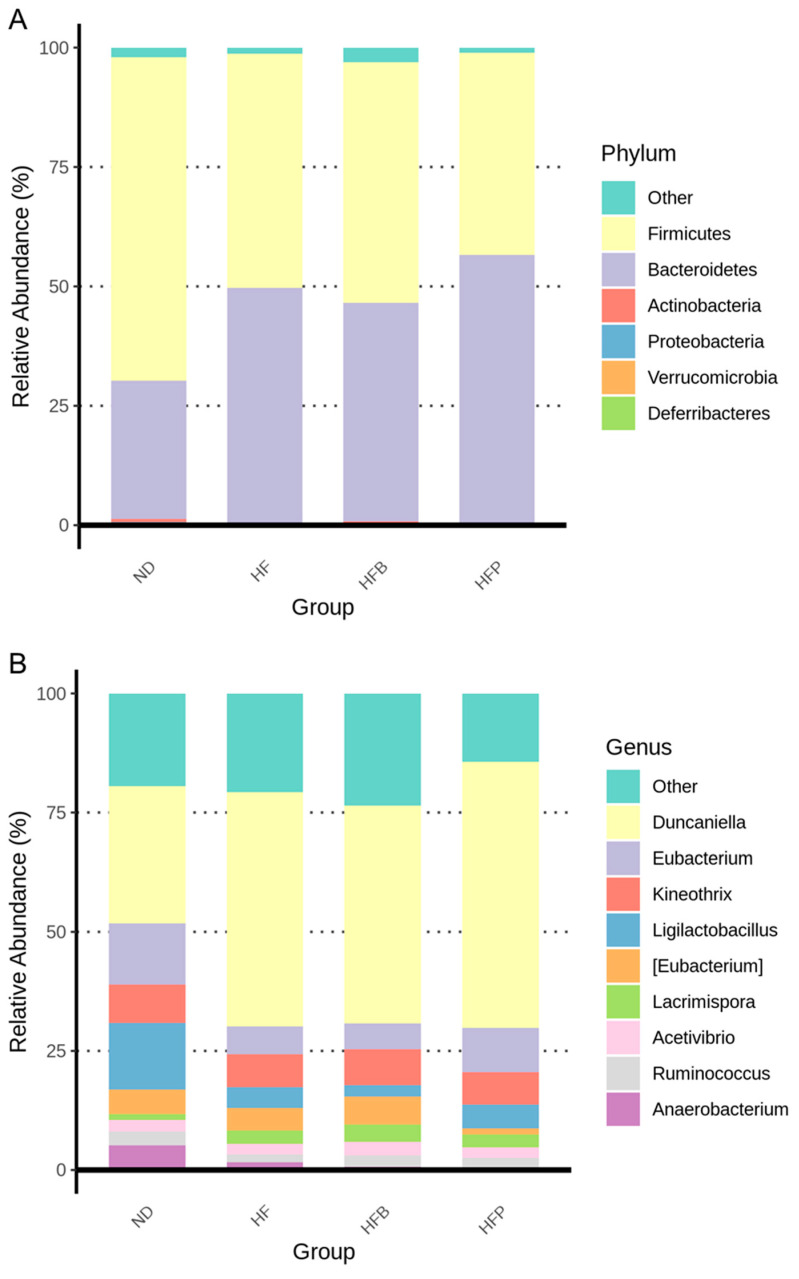
Relative abundance of dominant (**A**) phyla and (**B**) genera in the gut microbiota of male offspring at 12 weeks of age.

The microbiota composition of rat offspring at 12 weeks old is shown in Figure 5A at the phylum level. Firmicutes and Bacteroidetes are the dominant phyla among the four groups. This pattern closely resembled that seen in previous studies in rats [12,13,14,15]. Figure 5B reveals that *Duncaniella*, *Eubacterium*, *Kineothrix,* and *Ligilactobacillus* were the major genera in the four groups. 

**Figure 6:**
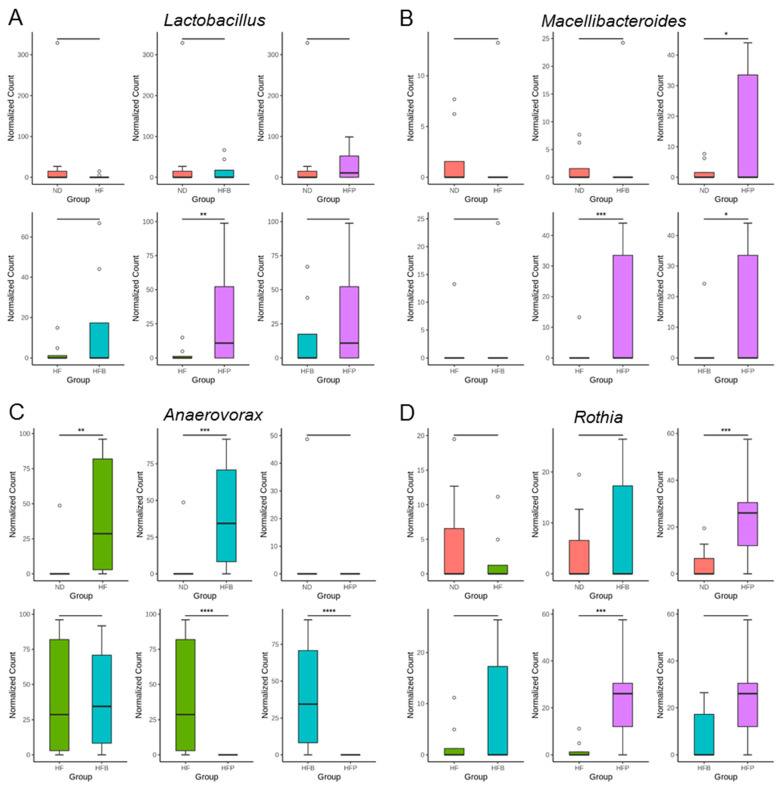
The comparison of genera (**A**) *Lactobacillus*, (**B**) *Macellibacteroides*, (**C**) *Anaerovorax*, and (**D**) *Rothia* between the ND (orange-red), HF (green), HFB (blue), and HFP (purple). *n* = 8/group. * *p* < 0.05; ** *p* < 0.01; *** *p* < 0.005; **** *p* < 0.001.

At the genus level, maternal propionate treatment altered gut microbiota composition in HFP offspring with a notable increase in *Lactobacillus* (Figure 6A). Similar patterns were observed with the genus *Macellibacteroides* (Figure 6B). Maternal HF consumption significantly increased the abundance of the genus *Anaerovorax* in the HF offspring compared with the ND group (Figure 6C). This increase was restored by propionate treatment but not by butyrate treatment. Additionally, maternal propionate treatment resulted in an increase in the abundance of genus *Rothia* in the HFP offspring in comparison to the ND and HF offspring (Figure 6D).

**Figure 7:**
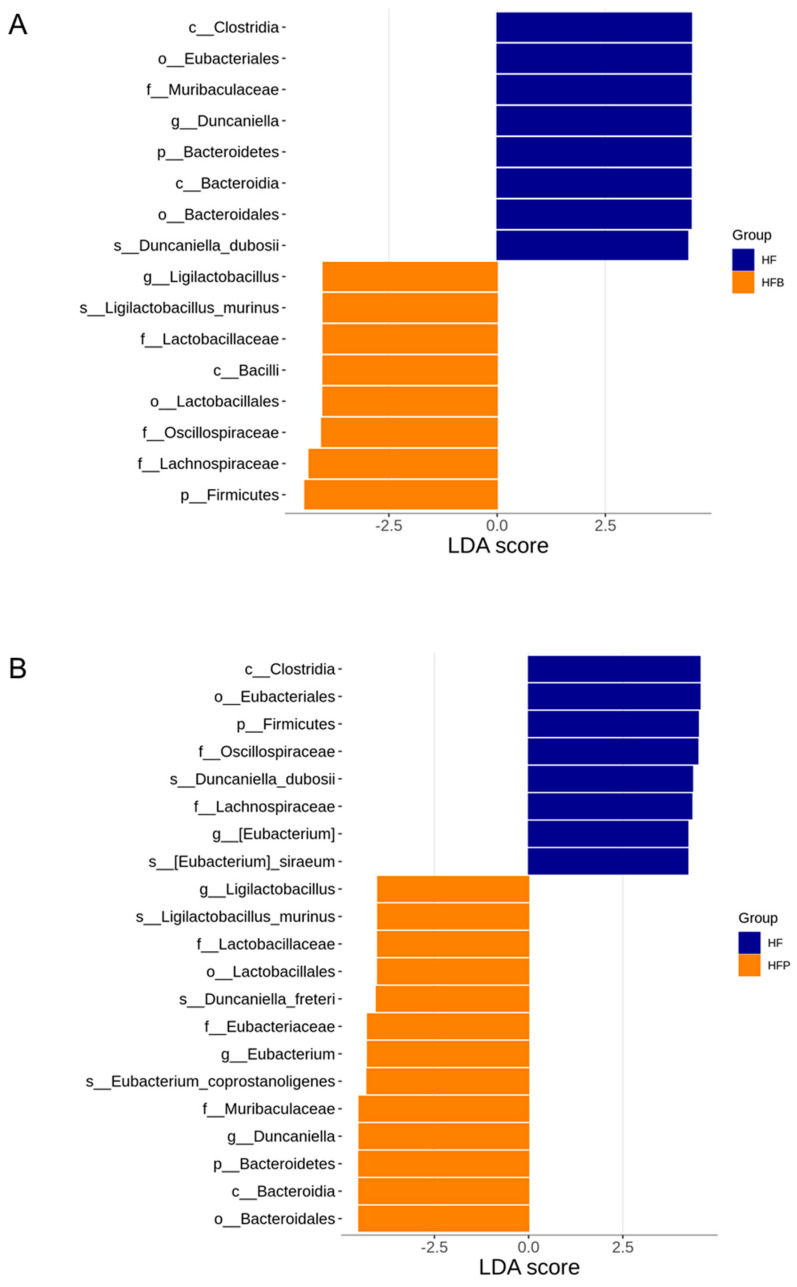
Linear discriminant analysis effect size (LEfSe) identified the most differentially abundant taxa between (**A**) the HF and HFB group and (**B**) the HF and HFP group. The threshold of the linear discriminant analysis score was set at 4.

The greatest differences in taxa abundance between the HF and HFB groups identified by the LEfSe analysis are shown in Figure 7A. In particular, the genus *Duncaniella* and the family and phylum to which it belongs were more abundant in the HF group. In contrast, maternal butyrate treatment caused a higher proportion of the genus *Ligilactobacillus*. Additionally, multiple levels of the most differentially abundant taxa between the HF and HFP offspring were illustrated in Figure 7B. HFP offspring had an increased abundance of *Ligilactobacillus*, *Eubacterium*, and *Duncaniella*.

### 3.5. NO and RAS

**Figure 8:**
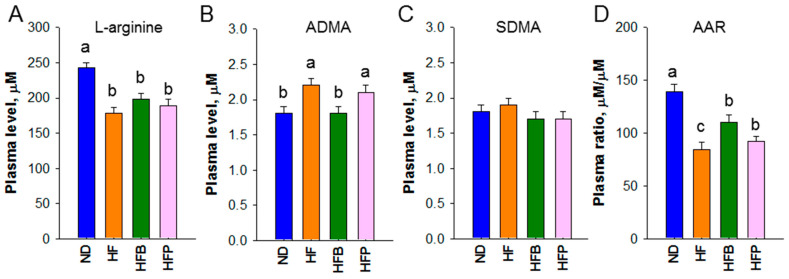
Plasma levels of (**A**) L-arginine, (**B**) asymmetric dimethylarginine (ADMA), (**C**) symmetric dimethylarginine (SDMA), and (**D**) L-arginine-to-ADMA ratio (AAR) of male offspring at age 12 weeks. *n* = 8/group; the letters represent the differences between the groups. Statistical analysis by one-way ANOVA, *p* < 0.05.

Since NO and the RAS participate in the regulation of BP, we further evaluated whether the protective effects of butyrate and propionate were associated with these mechanisms. As observed (Figure 8), maternal HF consumption caused a lower L-arginine and a higher ADMA level. This was accompanied by a lower plasma L-arginine-to-ADMA ratio in HF offspring compared with ND. Maternal butyrate treatment showed an improvement in plasma ADMA levels and the AAR. However, propionate supplementation had a negligible effect on NO parameters.

**Figure 9:**
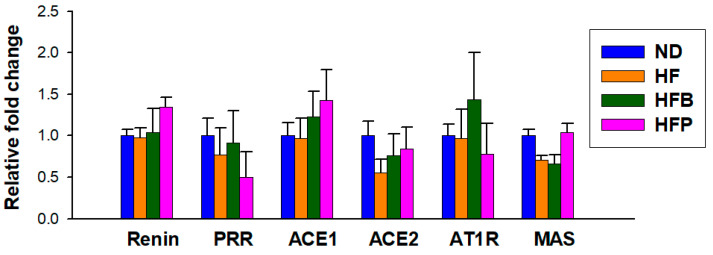
Renal mRNA expression of components belonging to the renin-angiotensin system of male offspring at age 12 weeks, including renin, (pro)renin receptor (PRR), angiotensin-converting enzyme-1 (ACE1) and -2 (ACE2), angiotensin II type 1 receptor (AT1R), and angiotensin (1–7) MAS receptor (MAS). *n* = 8/group.

We further analyzed the mRNA expression of RAS-related genes in offspring kidneys. As shown in Figure 9, no differences in the renal expression of RAS components between the four groups were seen.

## 4. Discussion

The present study appears to be the first to demonstrate that postbiotic supplementation with butyrate or propionate during gestation and lactation has the ability to protect adult offspring against hypertension induced by maternal high-fructose consumption. Our novel findings are specifically shown as follows: (1) Adult offspring born to dams fed with a high-fructose diet developed hypertension at age 12 weeks, which maternal butyrate or propionate supplementation prevented; (2) maternal butyrate treatment increased plasma concentrations of SCFA in adult offspring, despite no direct supplementation of butyrate by offspring; (3) maternal butyrate supplementation restored maternal HF diet-induced increase of TMAO, but caused a further decrease of DMA in the HFB offspring; (4) maternal propionate supplementation altered gut microbiota composition that directly reduced abundance of genus *Anaerovorax,* while increased genera *Lactobacillus*, *Macellibacteroides*, and *Rothia*; and (5) the beneficial action of propionate on maternal HF diet-primed offspring hypertension is linked to restoration of NO bioavailability.

The results of our study assessing maternal consumption of a fructose-rich diet are consistent with emerging human and experimental evidence that HF intake is linked to increased BP [3,4,5,20]. Several mechanisms have been proposed behind maternal HF diet-primed hypertension programming, such as an aberrant renin-angiotensin-aldosterone system (RAAS) [5], increased oxidative stress [21], deficient nitric oxide [22], impaired sodium transporters [23], epigenetic regulation [24], and dysregulated nutrient-sensing signals [21]. Our study further highlights the importance of gut microbiota and its derived metabolites in hypertension of developmental origins. 

With growing knowledge of the gut microbiome’s influence on hypertension control [25,26], modulation of the gut microbiota offers a novel therapeutic strategy to treat and prevent hypertension [27]. These gut microbiota-targeted therapies cover probiotics, prebiotics, postbiotics, fecal microbiota transplantation, bacterial metabolite modulation, etc. [27]. So far, probiotics, prebiotics, and TMAO modulation have shown benefits against maternal HF diet-primed offspring hypertension [14,28]. As far as we are aware, this is the first time to show that postbiotic supplementation with butyrate or propionate during gestation and lactation has the ability to protect against offspring hypertension in this model. Both postbiotics, butyrate and propionate, are currently under study as potential therapies for metabolic diseases [29]. Our results confirm prior research showing that butyrate and propionate have vasodilatory action [9,10], resulting in the reduction of BP in established and programmed hypertension models [12,13,14,30]. Maternal HF diet-induced hypertension would, however, be prevented by butyrate or propionate supplementation in different ways. 

The positive effects of butyrate treatment against maternal HF diet-primed offspring hypertension are related to increased SCFA concentrations, including butyric acid, isobutyric acid, propionic acid, and valeric acid. SCFAs modify BP in a manner that is differentially modulated by the actions of their SCFA receptors. The initiation of GPR109A and GPR41 can reduce BP. Conversely, they can be counteracted by Olfr78 and GPR43, resulting in vasoconstriction [9]. As butyric acid and isobutyric acid were more selective for GPR41, butyrate supplementation would, therefore, lead to a vasodilatory effect. 

Notably, results from our study reveal that butyrate supplementation also influences another microbial metabolite, TMAO. TMAO is dose-dependently associated with hypertension risk [31]. Reductions in plasma TMAO and its metabolite DMA are possibly attributed to decreased gut microbial TMA formation. The beneficial effects of butyrate supplementation against hypertension are, at least in part, associated with mediating TMAO metabolism.

Another protective effect of butyrate might involve its ability to increase NO bioavailability. Deficient in NO is a vital mechanism underlying hypertension of developmental origin [32]. Our current study found that butyrate supplementation decreases ADMA, an NOS inhibitor, along with increasing the AAR, an index of NO bioavailability. Considering that NO has vasodilative action, our data agree with previous work showing that butyrate can enhance NO production [33], which butyrate supplementation protected offspring against in this model.

According to our data, propionate supplementation increased the abundance of *Lactobacillus*, *Macellibacteroides*, and *Rothia* but decreased *Anaerovorax*. Up to date, their relationships with the developmental programming of hypertension are little known, especially in the maternal HF diet model. *Lactobacillus* spp. are considered beneficial bacteria with probiotic properties [34]. Our result was unsurprising in view of prior research showing the beneficial effects of *Lactobacillus* on hypertension [35]. A former study reported that a high abundance of the genus *Macellibacteroides* correlated with normal BP in humans [36]. Another report revealed that *Rothia* was negatively associated with hypertension, while *Anaerovorax* occurred in greater amounts in the hypertensive phenotype [37]. In support of previous work indicating that specific taxa were related to the development of hypertension, propionate supplementation reduced BP, coinciding with altered gut microbiota composition that enhanced or reduced specific microbes. Together, further studies are needed to determine whether propionate treatment protecting offspring against maternal HF diet-induced hypertension is directly relevant to alterations of these specific microbes. Similar to butyrate, propionate supplementation increased plasma concentrations of propionic acid, isobutyric acid, and valeric acid. Consistent with prior studies demonstrating these SCFAs have a BP-lowering effect [9,38], our study provides additional evidence that early supplementation with specific SCFAs could reverse adverse programming processes and provide benefits against programmed hypertension. 

Consistent with previous research [20,39], our study finds that maternal fructose consumption causes BW to increase in adult offspring. Previous animal studies demonstrate that treatment with SCFA can reduce or reverse gains in BW [40]. A previous study revealed that oral propionate administration prevented weight gain in overweight adult humans [41]. In line with this observation, we found maternal HF diet-induced offspring’s weight gain was improved by propionate supplementation. However, our current study did not detect the effect of butyrate treatment on offspring’s weight gain despite oral administration of sodium butyrate leading to a loss of BW in obese mice [42]. The mechanisms through which butyrate directly mediates BW in established obesity may be different from those involved in the developmental programming model. As the protective actions of each SCFA may be different, additional studies are required to explore their differences for future practical applications of SCFAs as postbiotics for HF diet-related diseases.

This study has some limitations. Firstly, this is a male-only study because hypertension appears earlier in males than in females. Whether sex differences are associated with responses to butyrate or propionate treatment deserves further clarification. Another limitation was that we mainly focused on the kidneys. The protective actions of both postbiotics on maternal HF diet-primed hypertension in adult offspring may be attributed to other BP-controlled organ systems. Third, we did not examine other microbial metabolites. Despite our study providing evidence for SCFAs and TMAO in this model of programming hypertension, the impact of other gut microbiota-derived metabolites is still unclear. Although several mechanisms (e.g., oxidative stress and aberrant RAAS) behind HF-induced hypertension programming have been proposed [20], we were unable to examine them all in the current study. Whether the positive effects of butyrate and propionate are attributed to other mechanisms is worth further study. Lastly, the gut microbiota profile was solely studied in adult progeny but not in dams. Comparing progeny to dams in a paired fashion might afford more details on how maternal microbiota impact the offspring.

## 5. Conclusions

In summary, we found that early-life supplementation with butyrate or propionate reprograms maternal HF diet-induced adverse programming processes in adult offspring. Although both postbiotics were similar in terms of protecting against offspring hypertension and increasing circulating SCFA concentrations, butyrate and propionate had differential protective mechanisms. Butyrate supplementation had a more profound impact on TMAO metabolism and the NO pathway, while propionate treatment, more specifically, influenced gut microbiota composition and specific microbes. Through our study, we demonstrated that alterations in maternal nutrition drove programming and reprogramming effects in adult offspring. This study not only supports findings that maternal HF intake may have an adverse impact on their offspring’s health in later life, but that early-life postbiotic supplementation may serve as a potential therapeutic strategy for the prevention of hypertension.

## Figures and Tables

**Table 1:** Primer sequences used for real-time quantitative PCR (qPCR) analysis.

Gene	Sense	Anti-Sense
GPR41	TCTTCACCACCGTCTATCTCAC	CACAAGTCCTGCCACCCTC
GPR43	CTGCCTGGGATCGTCTGTG	CATACCCTCGGCCTTCTGG
GPR109A	CGGTGGTCTACTATTTCTCC	CCCCTGGAATACTTCTGATT
Olfr78	GAGGAAGCTCACTTTTGGTTTGG	CAGCTTCAATGTCCTTGTCACAG
Renin	AACATTACCAGGGCAACTTTCACT	ACCCCCTTCATGGTGATCTG
PRR	GAGGCAGTGACCCTCAACAT	CCCTCCTCACACAACAAGGT
ACE1	CACCGGCAAGGTCTGCTT	CTTGGCATAGTTTCGTGAGGAA
AT1R	GCTGGGCAACGAGTTTGTCT	CAGTCCTTCAGCTGGATCTTCA
ACE2	ACCCTTCTTACATCAGCCCTACTG	TGTCCAAAACCTACCCCACATAT
MAS	CATCTCTCCTCTCGGCTTTGTG	CCTCATCCGGAAGCAAAGG
R18S	GCCGCGGTAATTCCAGCTCCA	CCCGCCCGCTCCCAAGATC

**Table 2:** Weights, BP, and the creatinine concentration of male offspring at 12 weeks of age.

Group	ND	HF	HFB	HFP
Mortality	0%	0%	0%	0%
Body weight (BW) (g)	291 ± 4 ^b^	331 ± 9 ^a^	341 ± 11 ^a^	308 ± 9 ^b^
Left kidney weight (g)	1.31 ± 0.02 ^b^	1.58 ± 0.04 ^a^	1.46 ± 0.07 ^a^	1.38 ± 0.04 ^b^
Left kidney weight/100 g BW	0.45 ± 0.01 ^a^	0.48 ± 0.01 ^a^	0.43 ± 0.01 ^b^	0.45 ± 0.01 ^a^
Systolic blood pressure (mmHg)	131 ± 1 ^b^	142 ± 2 ^a^	131 ± 1 ^b^	129 ± 1 ^b^
Diastolic blood pressure (mmHg)	92 ± 1 ^a^	95 ± 3 ^a^	89 ± 2 ^b^	87 ± 3 ^b^
Mean arterial pressure (mmHg)	105 ± 1 ^b^	111 ± 12 ^a^	103 ± 2 ^b^	101 ± 2 ^b^
Creatinine (μM/L)	12.1 ± 0.58	12.65 ± 0.96	11.39 ± 0.61	13.1 ± 0.54

*n* = 8/group; the letters represent the differences between the groups. Statistical analysis by a one-way ANOVA, *p* < 0.05.

**Table 3:** Plasma SCFA levels of male offspring at 12 weeks of age.

Group	ND	HF	HFB	HFP
Acetic acid (μM)	1261 ± 78	1028 ± 79	1249 ± 85	1081 ± 83
Propionic acid (μM)	8.3 ± 0.7 ^b^	6 ± 0.7 ^c^	18.2 ± 3.2 ^a^	27.4 ± 3 ^a^
Isobutyric acid (μM)	3 ± 0.2 ^a^	1 ± 0.3 ^b^	3.5 ± 0.4 ^a^	2.9 ± 0.5 ^a^
Butyric acid (μM)	11.1 ± 0.3 ^a^	10 ± 0.2 ^b^	14.7 ± 0.9 ^a^	10.8 ± 0.4 ^b^
Isovaleric acid (μM)	4.9 ± 0.1	4.8 ± 0.1	5.2 ± 0.1	4.9 ± 0.1
Valeric acid (μM)	9.2 ± 0.8 ^a^	0.8 ± 0.2 ^c^	7 ± 0.6 ^b^	4.7 ± 1 ^b^

*n* = 8/group; the letters represent the differences between the groups. Statistical analysis by a one-way ANOVA, *p* < 0.05.

## Data Availability

Data are contained within the article.

## References

[1] Hanson M. (2015). The birth and future health of DOHaD. J. Dev. Orig. Health Dis..

[2] Hsu C.N., Tain Y.L. (2018). The Double-Edged Sword Effects of Maternal Nutrition in the Developmental Programming of Hypertension. Nutrients.

[3] Madero M., Perez-Pozo S.E., Jalal D., Johnson R.J., Sánchez-Lozada L.G. (2011). Dietary fructose and hypertension. Curr. Hypertens. Rep..

[4] Tain Y.L., Chan J.Y., Hsu C.N. (2016). Maternal Fructose Intake Affects Transcriptome Changes and Programmed Hypertension in Offspring in Later Life. Nutrients.

[5] Seong H.Y., Cho H.M., Kim M., Kim I. (2019). Maternal High-Fructose Intake Induces Multigenerational Activation of the Renin-Angiotensin-Aldosterone System. Hypertension.

[6] Hsu C.N., Tain Y.L. (2020). Early-Life Programming and Reprogramming of Adult Kidney Disease and Hypertension: The Interplay between Maternal Nutrition and Oxidative Stress. Int. J. Mol. Sci..

[7] Al Rubaye H., Adamson C.C., Jadavji N.M. (2021). The role of maternal diet on offspring gut microbiota development: A review. J. Neurosci. Res..

[8] Hsu C.N., Yu H.R., Chan J.Y.H., Wu K.L.H., Lee W.C., Tain Y.L. (2022). The Impact of Gut Microbiome on Maternal Fructose Intake-Induced Developmental Programming of Adult Disease. Nutrients.

[9] Pluznick J.L. (2017). Microbial short-chain fatty acids and blood pressure regulation. Curr. Hypertens. Rep..

[10] Zółkiewicz J., Marzec A., Ruszczyński M., Feleszko W. (2020). Postbiotics-A step beyond pre- and probiotics. Nutrients.

[11] Ziętek M., Celewicz Z., Szczuko M. (2021). Short-Chain Fatty Acids, Maternal Microbiota and Metabolism in Pregnancy. Nutrients.

[12] Hsu C.N., Yu H.R., Lin I.C., Tiao M.M., Huang L.T., Hou C.Y., Chang-Chien G.P., Lin S., Tain Y.L. (2022). Sodium butyrate modulates blood pressure and gut microbiota in maternal tryptophan-free diet-induced hypertension rat offspring. J. Nutr. Biochem..

[13] Tain Y.L., Hou C.Y., Chang-Chien G.P., Lin S.F., Hsu C.N. (2022). Perinatal Propionate Supplementation Protects Adult Male Offspring from Maternal Chronic Kidney Disease-Induced Hypertension. Nutrients.

[14] Hsu C.N., Chang-Chien G.P., Lin S., Hou C.Y., Tain Y.L. (2019). Targeting on Gut Microbial Metabolite Trimethylamine-N-Oxide and Short-Chain Fatty Acid to Prevent Maternal High-Fructose-Diet-Induced Developmental Programming of Hypertension in Adult Male Offspring. Mol. Nutr. Food Res..

[15] Hsu C.N., Hou C.Y., Chang-Chien G.P., Lin S., Chan J.Y.H., Lee C.T., Tain Y.L. (2021). Maternal resveratrol therapy protected adult rat offspring against hypertension programmed by combined exposures to asymmetric dimethylarginine and trimethylamine-Noxide. J. Nutr. Biochem..

[16] Tain Y.L., Wu K.L.H., Lee W.C., Leu S., Chan J.Y.H. (2018). Prenatal Metformin Therapy Attenuates Hypertension of Developmental Origin in Male Adult Offspring Exposed to Maternal High-Fructose and Post-Weaning High-Fat Diets. Int. J. Mol. Sci..

[17] Reckelhoff J.F. (2001). Gender differences in the regulation of blood pressure. Hypertension.

[18] Bolyen E., Rideout J.R., Dillon M.R., Bokulich N.A., Abnet C.C., Al-Ghalith G.A., Alexander H., Alm E.J., Arumugam M., Asnicar F. (2019). Reproducible, interactive, scalable and extensible microbiome data science using QIIME 2. Nat. Biotechnol..

[19] Bode-Böger S.M., Scalera F., Ignarro L.J. (2007). The L-arginine paradox: Importance of the L-arginine/asymmetrical dimethylarginine ratio. Pharmacol. Ther..

[20] Lee W.C., Wu K.L.H., Leu S., Tain Y.L. (2018). Translational insights on developmental origins of metabolic syndrome: Focus on fructose consumption. Biomed. J..

[21] Tain Y.L., Lee W.C., Wu K.L.H., Leu S., Chan J.Y.H. (2018). Resveratrol Prevents the Development of Hypertension Programmed by Maternal Plus Post-Weaning High-Fructose Consumption through Modulation of Oxidative Stress, Nutrient-Sensing Signals, and Gut Microbiota. Mol. Nutr. Food Res..

[22] Tain Y.L., Leu S., Wu K.L.H., Lee W.C., Chan J.Y.H. (2014). Melatonin prevents maternal fructose intake-induced programmed hypertension in the offspring: Roles of nitric oxide and arachidonic acid metabolites. J. Pineal Res..

[23] Tain Y.L., Lee W.C., Leu S., Wu K., Chan J. (2015). High salt exacerbates programmed hypertension in maternal fructose-fed male offspring. Nutr. Metab. Cardiovasc. Dis..

[24] Tain Y.-L., Wu K.L., Lee W.-C., Leu S., Chan J.Y. (2015). Maternal fructose-intake-induced renal programming in adult male offspring. J. Nutr. Biochem..

[25] Muralitharan R.R., Jama H.A., Xie L., Peh A., Snelson M., Marques F.Z. (2020). Microbial Peer Pressure: The Role of the Gut Microbiota in Hypertension and Its Complications. Hypertension.

[26] Mishima E., Abe T. (2022). Role of the microbiota in hypertension and antihypertensive drug metabolism. Hypertens. Res..

[27] Tain Y.L., Hsu C.N. (2022). Hypertension of Developmental Origins: Consideration of Gut Microbiome in Animal Models. Biomedicines.

[28] Hsu C.N., Lin Y.J., Hou C.Y., Tain Y.L. (2018). Maternal Administration of Probiotic or Prebiotic Prevents Male Adult Rat Offspring against Developmental Programming of Hypertension Induced by High Fructose Consumption in Pregnancy and Lactation. Nutrients.

[29] Bourebaba Y., Marycz K., Mularczyk M., Bourebaba L. (2022). Postbiotics as potential new therapeutic agents for metabolic disorders management. Biomed. Pharmacother..

[30] Xu C., Marques F.Z. (2022). How Dietary Fibre, Acting via the Gut Microbiome, Lowers Blood Pressure. Curr. Hypertens. Rep..

[31] Ge X., Zheng L., Zhuang R., Yu P., Xu Z., Liu G., Xi X., Zhou X., Fan H. (2020). The Gut Microbial Metabolite Trimethylamine N-Oxide and Hypertension Risk: A Systematic Review and Dose-Response Meta-analysis. Adv. Nutr..

[32] Hsu C.N., Tain Y.L. (2019). Regulation of Nitric Oxide Production in the Developmental Programming of Hypertension and Kidney Disease. Int. J. Mol. Sci..

[33] Morikawa A., Sugiyama T., Koide N., Mori I., Mu M.M., Yoshida T., Hassan F., Islam S., Yokochi T. (2004). Butyrate enhances the production of nitric oxide in mouse vascular endothelial cells in response to gamma interferon. J. Endotoxin Res..

[34] Huang R., Wu F., Zhou Q., Wei W., Yue J., Xiao B., Luo Z. (2022). Lactobacillus and intestinal diseases: Mechanisms of action and clinical applications. Microbiol. Res..

[35] de Assis Gadelha D.D., de Brito Alves J.L., da Costa P.C.T., da Luz M.S., de Oliveira Cavalcanti C., Bezerril F.F., Almeida J.F., de Campos Cruz J., Magnani M., Balarini C.M. (2022). Lactobacillus group and arterial hypertension: A broad review on effects and proposed mechanisms. Crit. Rev. Food Sci. Nutr..

[36] Dan X., Mushi Z., Baili W., Han L., Enqi W., Huanhu Z., Shuchun L. (2019). Differential Analysis of Hypertension-Associated Intestinal Microbiota. Int. J. Med. Sci..

[37] Sun S., Lulla A., Sioda M., Winglee K., Wu M.C., Jacobs D.R., Shikany J.M., Lloyd-Jones D.M., Launer L.J., Fodor A.A. (2019). Gut Microbiota Composition and Blood Pressure: The CARDIA Study. Hypertension.

[38] Onyszkiewicz M., Gawrys-Kopczynska M., Sałagaj M., Aleksandrowicz M., Sawicka A., Koźniewska E., Samborowska E., Ufnal M. (2020). Valeric acid lowers arterial blood pressure in rats. Eur. J. Pharmacol..

[39] Saad A.F., Dickerson J., Kechichian T.B., Yin H., Gamble P., Salazar A., Patrikeev I., Motamedi M., Saade G.R., Costantine M.M. (2016). High-fructose diet in pregnancy leads to fetal programming of hypertension, insulin resistance, and obesity in adult offspring. Am. J. Obstet. Gynecol..

[40] Canfora E.E., Jocken J.W., Blaak E.E. (2015). Short-chain fatty acids in control of body weight and insulin sensitivity. Nat. Rev. Endocrinol..

[41] Chambers E.S., Viardot A., Psichas A., Morrison D.J., Murphy K.G., Zac-Varghese S.E., MacDougall K., Preston T., Tedford C., Finlayson G.S. (2015). Effects of targeted delivery of propionate to the human colon on appetite regulation, body weight maintenance and adiposity in overweight adults. Gut.

[42] Gao Z., Yin J., Zhang J., Ward R.E., Martin R.J., Lefevre M., Cefalu W.T., Ye J. (2009). Butyrate improves insulin sensitivity and increases energy expenditure in mice. Diabetes.

